# Prolonged tedizolid use in cutaneous non-tuberculous mycobacterial infection

**DOI:** 10.1016/j.jctube.2021.100261

**Published:** 2021-07-14

**Authors:** Timothy David Shaw, Mark Smyth, Graham Turner, Michael Hunter

**Affiliations:** a Wellcome-Wolfson Institute for Experimental Medicine, Queen’s University Belfast, Belfast, UK; b Department of Medical Microbiology, Belfast Health and Social Care Trust, Belfast, UK; c Gastroenterology Unit, Belfast Health and Social Care Trust, Belfast, UK; d Infectious Diseases Unit, Belfast Health and Social Care Trust, Belfast, UK

**Keywords:** Non-tuberculous mycobacteria, *Mycobacterium chelonae*, Extrapulmonary infection, Tedizolid

## Abstract

Cutaneous non-tuberculous mycobacterial (NTM) infections are an emerging infectious disease and require a protracted course of combination antibiotics. Antimicrobial choice is limited by resistance and toxicity. Tedizolid is a new oxazolidinone antibiotic with *in vitro* activity against some NTM, but its use in the management of extrapulmonary NTM has not been described. We report on the utility of prolonged tedizolid use (8 months), after linezolid intolerance, in combination therapy in a case of difficult *Mycobacterium chelonae* cutaneous infection. Although tedizolid contributed to clinical remission, it may have also contributed to a multifocal peripheral neuropathy. Its efficacy may also have been limited by continued immunosuppression, with evidence to suggest disease relapse or treatment failure after completion of combination therapy. Tedizolid can be considered, with caution, in combination therapy for difficult cases of cutaneous NTM infection.

## 1 Introduction

Extrapulmonary non-tuberculous mycobacterial (NTM) infections affect a variety of sites, though cutaneous infections are most frequently reported [1]. Cutaneous NTM infections typically require a protracted course of antimicrobials and clinicians have a limited choice of agents [2]. Oxazolidinones achieve excellent penetration into skin/soft tissue and have *in vitro* activity against some non-tuberculous mycobacteria (NTM) [3]. However, prolonged use of linezolid is poorly tolerated due to haematological disorders and peripheral neuropathy.

Tedizolid is a newer oxazolidinone which has shown similar efficacy to linezolid in acute bacterial skin and soft tissue infections (ABSSIs), with reduced adverse side effects [4]. Its use in mycobacterial infections is limited by its absence in standard antimicrobial susceptibility testing (AST). Nevertheless, extended use of tedizolid has been described in small mycobacterial case series, compromising mostly pulmonary NTM infection [5], [6] and pulmonary tuberculosis [7]. Its use in extrapulmonary NTM infection is remains poorly characterized. We describe the extended use (8 months) of tedizolid, after linezolid toxicity, which contributed in combination therapy to remission for a patient with cutaneous *Mycobacterium chelonae* infection.

## 2 Case presentation

A male patient in his seventies was diagnosed with type 2 refractory coeliac disease with ulcerative jejunoileitis. He was commenced on mycophenolate after treatment failure with steroids and azithromycin. Four weeks later, he developed numerous erythematous skin lesions across all four limbs. Microscopy on skin biopsies revealed a few acid-fast bacilli (AFB) per field. Nucleic acid amplification testing was negative for *Mycobacterium tuberculosis* complex (Cepheid Xpert MTB/RIF). The isolate was detected in liquid media after 10 days (BD Bactec MGIT system) and grew on solid pyruvate Löwenstein–Jensen media. 16S rRNA analysis indicated a 99% sequence homology to *Mycobacterium chelonae* and *Mycobacterium abscessus.* Line probe assay confirmed mycobacterial identification as *M. chelonae.* AST was performed by a microdilution method according to Clinical Laboratory Standards Institute (CLSI) guidelines [8] and revealed the isolate had susceptibility to clarithromycin (0.5 mg/L) and tobramycin (2 mg/L); intermediate susceptibility to linezolid (16 mg/L) and amikacin (32 mg/L); and resistance to co-trimoxazole (>8/152 mg/L), ciprofloxacin (>4mg/L), moxifloxacin (8 mg/L), doxycycline (>16 mg/L) and cefoxitin (128 mg/L) (Table 1).

**Table 1:** Antimicrobial susceptibility testing results of *Mycobacterium chelonae* isolated from the patient’s skin biopsy.

Antimicrobial	MIC (mg/L)	Susceptibility
Amikacin	32	I
Cefoxitin	128	R
Ciprofloxacin	>4	R
Clarithromycin	0.5	S
Co-trimoxazole	>8/152	R
Doxycycline	>16	R
Linezolid	16	I
Moxifloxacin	8	R
Tobramycin	2	S

S: susceptible; I: intermediate susceptibility; R: resistant.

The patient was diagnosed with cutaneous *M. chelonae* infection in the setting of immunosuppression. His steroid dose was reduced and mycophenolate therapy discontinued. The patient was commenced empirically on oral clarithromycin, IV tobramycin and IV imipenem/cilastatin. This was rationalised after 5 weeks with AST results to oral clarithromycin, IV tobramycin and empirical IV tigecycline.

**Fig. 1:**
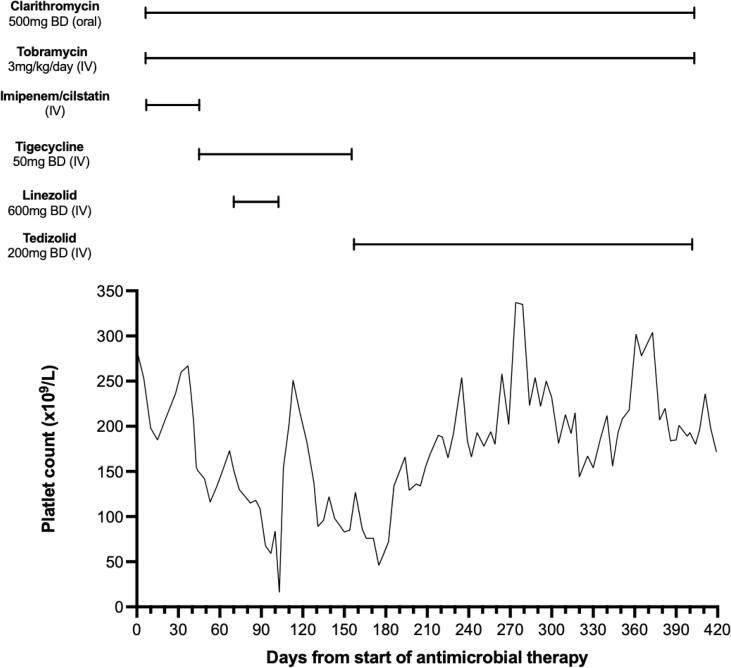
The patient’s platelet count during antimicrobial therapy. Linezolid commenced on day 70, after no improvement in cutaneous appearances. Platelets dropped to 16 × 10^9^/L within 30 days and linezolid was stopped. Tedizolid was commenced on day 160. After a mild, transient fall, platelet counts stabilised to a baseline of 130–300 × 10^9^/L and tezidolid was continued for 8 further months.

Baseline platelets levels were 200–250 × 10^9^/L and fell after adding tigecycline, though they remained in an acceptable and tolerable range (100–150 × 10^9^/L) (Fig. 1). After 10 weeks, linezolid was added as there was no improvement in the cutaneous lesions. Regression of skin lesions was observed but severe thrombocytopenia developed (platelet count 16 × 10^9^/L) and linezolid was stopped after 30 days. The patient regained baseline platelet levels within two weeks but the skin lesions re-appeared five weeks after stopping linezolid. Eight weeks after cessation of linezolid, tedizolid (200 mg IV OD) was commenced and tigecycline was stopped.

The skin lesions regressed following the addition of tedizolid and remained in remission until the end of his course. After an initial, transient fall (to 46 × 10^9^/L), platelet counts stabilized to a baseline of 130–300 × 10^9^/L (Fig. 1). The patient completed an 8-month course of tedizolid in combination with clarithromycin and tobramycin and was discharged off antibiotics. Tobramycin therapeutic drug monitoring continued at least weekly throughout the course, with most post-dose peaks reported in the range 5–10 mg/L and trough levels below 2 mg/L. After discharge, his immunosuppression was maintained on low-dose prednisolone only (7.5 mg once daily).

Although the combination of tedizolid, clarithromycin and tobramycin was efficacious, the patient developed some adverse neurological side effects. During inpatient treatment, he acquired irreversible bilateral sensorineural hearing loss (most likely caused by protracted aminoglycoside therapy). He also developed a right foot drop and nerve conduction studies revealed a length-dependent mixed axonal loss peripheral neuropathy. Over the three months following discharge and cessation of all antibiotics, he developed neuropathic pain in both feet.

The patient reported occasional, milder recurrence of similar skin lesions over the following year. Histological examination revealed necrotising granulomas with no evidence for AFB on staining, and mycobacterial culture was negative. The aetiology and nature of these lesions was unclear, but they were self-limiting and no further anti-mycobacterial therapy was given. The patient died over two years later from small bowel lymphoma as a complication of ulcerative jejunoileitis.

## 3 Discussion

Extrapulmonary NTM infection is an emerging infectious disease, typically associated with significant systemic illness, impaired immunity and/or structural abnormality in the infected organ [2]. Although cutaneous infection is most commonly reported, pathogenic NTM are being increasingly isolated from other sites including lymph nodes and the gastrointestinal tract [1]. Consequently, a diverse range of specialists including dermatologists, hematologists, gastroenterologists and surgeons are encountering cases more frequently.

Clinical practice guidelines for extra-pulmonary NTM infections are less comprehensive than those for pulmonary disease and are lacking an evidence base. This is exacerbated further by inter-species heterogeneity in growth rates, antimicrobial susceptibility and geographical distribution [2]. The latest consensus ATS statement guidelines for extra-pulmonary NTM infections (2007) limit treatment recommendations to 6–12 month course of three or more drugs with *in vitro* activity [9]. Therefore clinical advice sought from infection specialists is heavily reliant on obtainable antimicrobial susceptibility testing, expert opinion and reported case series.

Clinicians and patients must weigh the risks and benefits of using novel antimicrobials together, but published experience of prolonged tedizolid use in clinically-vulnerable patients is lacking. A small retrospective study reported on the safety and tolerability of prolonged tedizolid use (median 101 days) in 24 patients with NTM infection, of whom four had disseminated disease [6]. Peripheral neuropathy was the most common reported side effect (5 patients), though this was not described in relation to duration of therapy or disseminated infection in the report. Others have reported tedizolid use in a case of pulmonary NTM disease after linezolid-induced nausea and progressive anemia [5]. Their patient tolerated 58 days of tedizolid, before treatment was stopped due to a fall in haemoglobin. The same group later reported a 20-month course of tedizolid was safe and efficacious in an adolescent liver transplant recipient with pulmonary tuberculosis [7]. To our knowledge, this the first detailed report of prolonged tedizolid use in extra-pulmonary NTM infection.

In our case, tedizolid was used for 8 months in an older patient with substantial co-morbidities, including ulcerative jejunoileitis requiring immunosuppression and parenteral nutrition. The addition of oxazolidinones to combination therapy was strongly associated with remission of cutaneous infection, and tedizolid did not cause the same thrombocytopenia as linezolid. The recurrence of multifocal necrotizing granulomatous skin lesions after treatment completion, though negative on microscopy and culture, was concerning for treatment failure or disease relapse. Fortunately, these were milder and self-limiting which was likely related to the reduced dependence on immunosuppressant therapy. This highlights the importance of promoting adequate host immunity in managing extrapulmonary NTM infection and warrants caution for reliance on antimicrobial therapy alone.

The patient acquired neurological deficits during therapy which were likely caused by a combination of factors. The lower limb motor deficit and neuropathic pain is consistent with reports of peripheral neuropathy arising from prolonged linezolid and tedizolid therapy in the literature [6], [10]. It is possible that there was a cumulative effect of oxazolidinone therapy, combining the 8-month tedizolid course with 3-weeks prior exposure to linezolid. Tedizolid has compared favourably to linezolid in studies of ABSSIs [11]. It had non-inferior efficacy and reduced risk of some side effects, including thrombocytopenia, though it is important to note these studies tested short-term therapy (less than 14 days). Dose reduction of linezolid in long-term use has been described (i.e. from 600 mg BD to OD) but is still associated with thrombocytopenia [12]. Other contributing factors in this case included nutritional deficiencies relating to intestinal failure. These are associated with several forms of peripheral neuropathy, even in the setting of parenteral nutrition [13], [14]. It is also possible he developed a critical care neuropathy which, though observed frequently in intensive care, is not as well-characterised in long-term inpatients outside the ICU [15].

One further limitation to tedizolid use is the availability of susceptibility testing on clinical NTM isolates. A recent report on antimicrobial susceptibility testing of tedizolid against NTM included two isolates of *M. chelonae* [3]*.* Tedizolid was found to have MICs of 0.5–1 mg/L against *M. chelonae* and synergistic activity in combination with clarithromycin, cefoxitin, tigecycline or amikacin. However, tedizolid susceptibility does not extrapolate directly from linezolid susceptibility, which is currently the only oxazolidinone with a CSLI breakpoint for NTMs. For this reason, tedizolid MICs are typically not included in the AST for NTMs at reference laboratories. This may change with time, as tedizolid is gaining recognition as an attractive alternative to linezolid for broader NTM *in vitro* activity and reduced toxicity.

## 4 Conclusion

In summary, we report a case of multifocal cutaneous *M. chelonae* in a clinically-vulnerable patient which was not controlled with combination antimicrobial therapy until an oxazolidinone was added. Tedizolid was better tolerated than linezolid in terms of adverse haematological side effects but may have contributed to peripheral neuropathy. The patient had clinical remission on completion of antimicrobials, but the recurrence of necrotizing granulomatous skin lesions, though culture negative, raises the possibility of disease relapse or treatment failure. Tedizolid can be considered, with caution, in combination therapy for difficult extrapulmonary NTM infections.

## Funding

This report did not receive any specific grant from funding agencies. MS, GT and MH conducted this report as part of their routine work in the Belfast Health and Social Care Trust. TS is supported by an MRC Clinical Research Training Fellowship (MR/R017867/1) and has a joint contract with Queen’s University Belfast and the Belfast Health and Social Care Trust.

## Declaration of competing interests

The authors have no known competing interests to declare.

## Ethical statement

The patient whose case is described has died. The authors are grateful to the patient’s next-of-kin who have given written consent for this report to be published.

## Author contribution

All authors meet the authorship criteria. G.T. and M.H. were responsible clinicians for the patient. M.S. was responsible for laboratory testing and reporting. T.S. drafted the manuscript and assisted in clinical management. All authors read, revised and approved the final manuscript.
